# *BRAF* V600E and *SRC* mutations as molecular markers for predicting prognosis and conversion surgery in Stage IV colorectal cancer

**DOI:** 10.1038/s41598-019-39328-6

**Published:** 2019-02-21

**Authors:** Yoshifumi Shimada, Yusuke Muneoka, Masayuki Nagahashi, Hiroshi Ichikawa, Yosuke Tajima, Yuki Hirose, Takuya Ando, Masato Nakano, Jun Sakata, Hitoshi Kameyama, Yasumasa Takii, Yiwei Ling, Shujiro Okuda, Kazuaki Takabe, Toshifumi Wakai

**Affiliations:** 1 Division of Digestive and General Surgery, Niigata University Graduate School of Medical and Dental Sciences, Niigata, Japan; 2 Department of Surgery, Niigata Cancer Center Hospital, Niigata, Japan; 3 Division of Bioinformatics, Niigata University Graduate School of Medical and Dental Sciences, Niigata, Japan; 4 Division of Breast Surgery, Roswell Park Comprehensive Cancer Center, Elm & Carlton Streets, Buffalo, NY 14263 USA; 5 Department of Surgery, University at Buffalo Jacobs School of Medicine and Biomedical Sciences, The State University of New York, Buffalo, NY USA; 6 Department of Breast Surgery and Oncology, Tokyo Medical University, Tokyo, Japan; 7 Department of Surgery, Yokohama City University, Yokohama, Japan

## Abstract

Comprehensive genomic sequencing (CGS) enables us to detect numerous genetic alterations in a single assay. We aimed to identify molecular markers for predicting prognosis and conversion surgery in Stage IV colorectal cancer (CRC) using CGS. One-hundred eleven patients with Stage IV CRC who underwent primary tumor resection were analyzed. We retrospectively investigated genetic alterations using CGS of a 415-gene panel. Clinicopathological variables and genetic alterations were analyzed to identify independent prognostic factors of overall survival (OS). Forty-five of 111 patients had R0 resection; of these, 11 patients underwent conversion surgery. Univariate and multivariate analyses identified histopathological grade 3, R0 resection, *BRAF* V600E mutation, and *SRC* mutation as independent prognostic factors for OS (*P* = 0.041, *P* = 0.013, *P* = 0.005, and *P* = 0.023, respectively). *BRAF* V600E and *SRC* mutations were mutually exclusive, and *SRC* mutation was significantly associated with left-sided tumor and liver metastasis compared to *BRAF* V600E mutation (*P* = 0.016 and *P* = 0.025, respectively). Eleven of the 74 initially unresectable patients underwent conversion surgery for R0 resection, yet none harbored *BRAF* V600E or *SRC* mutations. *BRAF* V600E and *SRC* mutations are important molecular markers which can predict prognosis and conversion surgery in Stage IV CRC.

## Introduction

Worldwide, colorectal cancer (CRC) was responsible for an estimated 1.4 million new cases and 694,000 deaths in 2012, and ranks as third most frequent cancer in men (after lung and prostate), and second in women (after breast)^1^. Despite widespread early detection screening for CRC, approximately 25% of patients with CRC are found to have distant metastases at time of diagnosis^2,3^. The American Joint Committee on Cancer (AJCC) defines Stage IV CRC as any tumor with an M stage of M1a, M1b, or M1c, which describes a tumor that has spread to distant organs, nodes or the peritoneum^2^. Stage IV CRC however, is a highly diverse disease, and as such, a more precise stratification of patients is required. Incorporation of non-anatomic factors beyond TNM would provide a more accurate and probabilistic individualized outcome prediction for precision medicine^4,5^.

In the past few years, there has been an explosion in the understanding of molecular markers. *KRAS*, *NRAS*, and *BRAF* are important components of the MEK/ERK pathway, which controls cell proliferation and survival in CRC. Activating somatic mutations at *KRAS*, *NRAS*, and *BRAF*, which predict poor response to anti-EGFR therapy^6,7^, are detected in up to 40%, 7%, and 10% patients with CRC, respectively^2,3,8^. Among them, *BRAF* V600E mutations are associated with a worse prognosis^9^, and are recognized as a non-anatomic poor prognostic factor in CRC^2^. The AJCC 8^th^ edition states that these non-anatomic factors are important to consider when making treatment decisions^2^.

R0 resection, a microscopically margin-negative resection where no gross or microscopic tumor remains, has been the most effective surgical treatment strategy in stage IV CRC^3,10^. For patients with oligometastatic disease contained to a single or a few organs, long-term survival or even cure can be attained in 20–50% patients following R0 resection of both primary and metastatic lesions^10^. Furthermore, there is the benefit of conversion surgery, where systemic therapy in patients with initially unresectable distant metastasis provides the prospect of R0 resection^3,11,12^. However, to date, predictive molecular markers for conversion surgery is not known.

SRC is a member of a superfamily of membrane-associated non-receptor protein tyrosine kinases^13^. These proteins are activated by a number of receptors, such as platelet-derived growth factors, epidermal growth factor, and fibroblast growth factor; and regulate a cascade of downstream targets to affect proliferation, adhesion, differentiation, and migration^14^. In CRC, a few reports have demonstrated that overexpression of SRC is associated with distant metastasis^14–16^ and drug resistance^17,18^; however, to date, the clinicopathological characteristics and clinical significance has not been fully elucidated.

Comprehensive genomic sequencing (CGS) is an emerging technology that can detect numerous genetic mutations and copy number alterations in a single assay. By utilizing CGS technology, projects such as The Cancer Genome Atlas (TCGA) have profiled genomic changes in many cancers including CRC^8^. Similarly, we have previously generated a genomic overview of Japanese CRC patients using a 415-gene CGS panel^19–21^, and speculated that CGS can detect clinically important genetic alterations of Stage IV CRC. We aimed to identify molecular markers for predicting prognosis and conversion surgery in Stage IV CRC using CGS.

## Materials and Methods

### Patients

**Figure 1:**
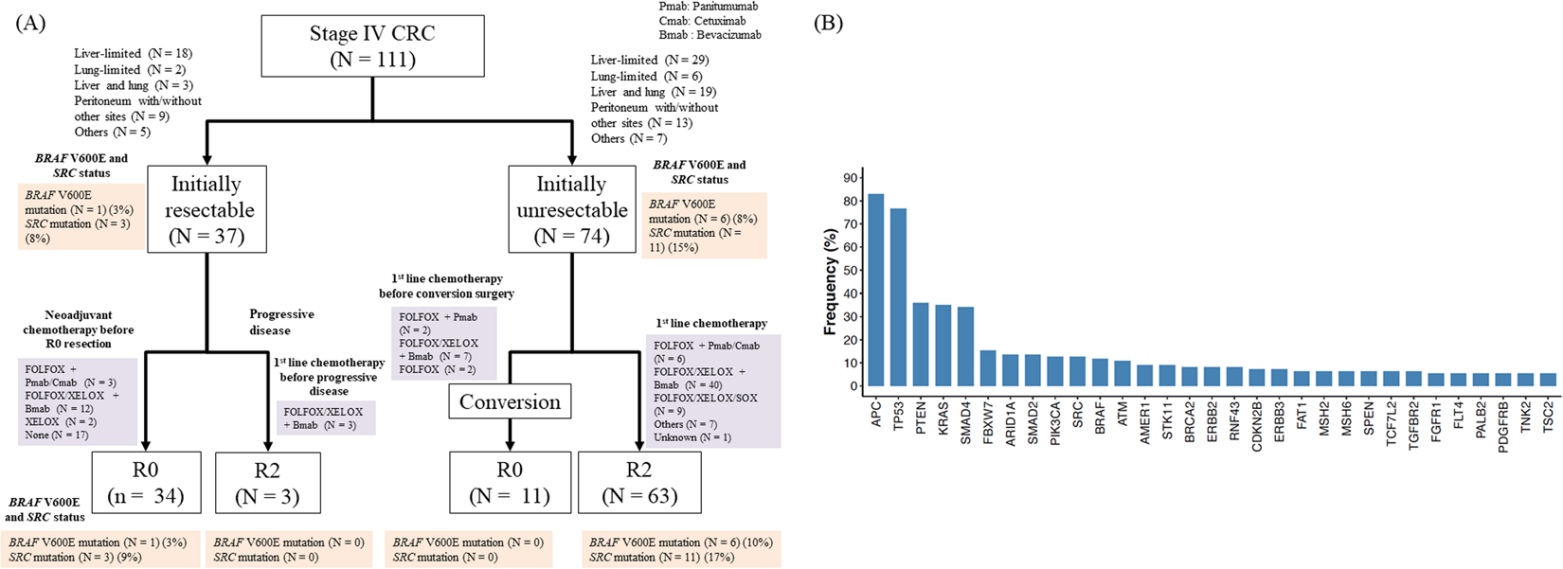
Overview of the 111-patient cohort with initial assessment of distant metastasis and residual tumor status (**A**). Genes (n = 31) altered in more that 5% of patients (**B**).

This retrospective analysis was performed in accordance with the Helsinki Declaration, and the Ethics Committee of the School of Medicine, Niigata University, approved the study protocol. All methods were performed in accordance with the relevant guidelines and regulations, and written informed consent was obtained from all patients. A total of 111 patients diagnosed with stage IV CRC (AJCC, 7^th^ edition)^22^ who underwent a primary tumor resection between 2009 and 2015 at the Niigata University Medical and Dental Hospital or Niigata Cancer Center Hospital were randomly selected, and enrolled. Patients with familial adenomatous polyposis or inflammatory bowel disease were excluded. Of the 111 patients, metastasis to the liver, lung, peritoneum, and other sites at initial assessment were identified in 88, 34, 22, and 23 patients, respectively. Thirty-seven and 74 patients were diagnosed with resectable and unresectable metastatic disease, respectively, at initial assessment (Fig. 1A). Resectability of metastatic disease was determined by colorectal and hepatobiliary surgeons of each of the two hospitals using computed tomography and/or magnetic resonance imaging.

### Residual tumor status and conversion surgery

Patients were classified according to residual tumor status. Patients who achieved R0 resection of both the primary lesion and distant metastasis were classified as “R0”, while patients for whom R0 resection could not be achieved were classified as “R2”. Conversion surgery was defined as R0 resection after systemic therapy for initially unresectable distant metastasis^3,11,12^.

### CGS analysis of primary tumors

As we previously reported, CGS of primary tumor was performed as follows^19–21^. Tumor content was evaluated by an independent pathologist for each sample using hematoxylin and eosin-stained slides to ensure >50% tumor content. Where applicable, unstained slides were macro-dissected to enrich for tumor content, and DNA was extracted using a BioStic FFPE Tissue DNA Isolation Kit (Mo Bio Laboratories, Inc., Carlsbad, CA). All sample preparations, CGS, and analytics were performed in a CLIA/CAP-accredited laboratory (KEW Inc., Cambridge, MA). DNA fragment (50–150 ng) libraries were prepared and enriched for the CancerPlex 415-gene panel (KEW Inc.)^19–21^. Sequencing was performed on Illumina MiSeq and NextSeq platforms with an average 500X sequencing depth. Genomic data were then processed through a proprietary bioinformatics platform and knowledgebase to identify multiple classes of genomic abnormalities, including single nucleotide substitutions, small insertions/deletions, copy number variations, and translocations. Single nucleotide variant (SNV) and insertion or deletion (indel) calling were only performed in genomic regions intended to be captured by the assay (region of interest). We set a standard threshold of 10% allelic fraction for calling SNVs and indels to focus on primary truncal driver mutations and avoid subclonal events. Copy number variants were called for exons as well as globally. We segmented regions using a Fused-Lasso method and exported results to a VCF file. The threshold for gain was >2.5 fold, and for loss was <0.5 fold. Variants were filtered or flagged according to technical quality (e.g. coverage, allelic fraction, number of supporting reads), presence in previously characterized normal samples, or presence/absence in the following databases: dbSNP, ExAC, COSMIC, ClinVar, and KEW. SNVs and indels in VCF format were annotated using SnpEff and the output was adapted according to HGVS recommendations. Tumors were tested for the presence of microsatellite instability (MSI) based on an extended loci panel. In addition to the Bethesda panel, a collection of 950 regions consisting of tandem repeats of one, two, or three nucleotides of minimum length of 10 bases were examined. The number of indels within the regions of interest was calculated, and tumors were classified as MSI-high (MSI-H) or microsatellite stable (MSS).

### Prognostic factors

To identify factors influencing overall survival (OS) after surgery, genetic alterations (identified using the 415 gene panel) and 15 clinicopathological variables (Table 1) were tested in all 111 patients. In this study, genetic alterations that occurred at a frequency of more than 5% were evaluated for their prognostic impact in univariate and multivariate analyses of 5-year OS. Regarding *BRAF* mutation, we separately evaluated *BRAF* V600E and non-V600E.

**Table 1:** Univariate and multivariate analyses of clinicopathological characteristics and genetic alterations on overall survival.

Variable	Modality	N (%)	Univariate		Multivariate	
			5-y OS %	*P*-value	HR (95% CI)	*P*-value
Age (years)	<65	62 (56)	9.3	0.604		
	≥65	49 (44)	15.2			
Sex	Male	66 (59)	5.0	0.104		
	Female	45 (41)	24.0			
Tumor size (mm)	<50	36 (32)	0.0	0.258		
	≥50	75 (68)	14.1			
Pre-operative CEA (ng/ml)	<200	85 (77)	13.8	0.338		
	≥200	26 (23)	5.1			
Primary tumor site	Right-sided	34 (31)	9.7	0.157		
	Left-sided	77 (69)	13.0			
Histopathological grade	G1, 2	78 (70)	14.2	**0.001**	1.00	
	G3	33 (30)	6.7		1.30 (1.01–1.68)	**0.041**
T category	T2, 3	47 (42)	0.0	0.059		
	T4	64 (58)	10.4			
N category	N0	15 (14)	24.1	**0.036**		
	N1	96 (86)	9.6			
M category	M1a	56 (50)	15.6	0.511		
	M1b	55 (50)	9.8			
Liver metastasis	Absent	23 (21)	21.8	0.139		
	Present	88 (79)	8.7			
Lung metastasis	Absent	77 (69)	11.8	0.824		
	Present	34 (31)	12.2			
Peritoneal metastasis	Absent	89 (80)	13.5	0.116		
	Present	22 (20)	5.5			
Liver-limited metastasis	Absent	64 (58)	11.9	0.815		
	Present	47 (42)	11.6			
Number pf metastatic sites	1	64 (58)	16.0	0.753		
	≥2	47 (42)	8.7			
Residual tumor	R0	45 (41)	19.2	**<0.001**	0.54 (0.33–0.88)	**0.013**
	R2	66 (59)	6.5		1.00	
*APC*	Wild-type	19 (17)	19.1	0.399		
	Mutant	92 (83)	9.5			
*ARID1A*	Wild-type	96 (86)	11.7	0.725		
	Mutant	15 (14)	16.1			
*ATM*	Wild-type	99 (89)	11.4	0.676		
	Mutant	12 (11)	11.1			
*BRAF*	Wild-type	98 (88)	12.7	**<0.001**	1.00	
	Non-V600E	6 (5)	33.3		1.52 (0.54–4.30)	0.424
	V600E	7 (6)	0.0		3.47 (1.45–8.29)	**0.005**
*FBXW7*	Wild-type	94 (85)	9.3	0.603		
	Mutant	17 (15)	25.7			
*KRAS*	Wild-type	72 (65)	12.0	0.348		
	Mutant	39 (35)	13.5			
*PIK3CA*	Wild-type	97 (87)	12.8	0.082		
	Mutant	14 (13)	0.0			
*PTEN*	Wild-type	71 (64)	15.9	0.677		
	Mutant	40 (36)	7.4			
*SMAD2*	Wild-type	96 (86)	12.9	0.288		
	Mutant	15 (14)	6.7			
*SMAD4*	Wild-type	73 (66)	15.3	0.281		
	Mutant	38 (34)	7.3			
*SRC*	Wild-type	97 (87)	14.2	**0.035**	1.00	
	Mutant	14 (13)	0.0		1.99 (1.10–3.59)	**0.023**
*TP53*	Wild-type	26 (23)	17.5	0.483		
	Mutant	85 (77)	10.3			

Only genes altered in more than 10% of patients (n = 12) are noted in this table, but all 31 genes altered in more than 5% of patients were evaluated.

*95% CI* 95% confidence interval, *HR* hazard ratio, *OS* overall survival.

### Genetic alterations in EGFR pathway

To investigate the association between efficacy of anti-EGFR therapy and genetic alterations in EGFR pathway, genetic alterations of TK receptors (*ERBB2*, *MET*, *EGFR*, *FGFR1*, and *PDGFRA*), MEK/ERK pathway (*KRAS*, *NRAS*, *HRAS*, *BRAF*, and *MAPK2K1*), and PI3K pathway (*PTEN* and *PIK3CA*) were analyzed using CGS of the 415-gene panel. We defined patients who had no alterations in any of the 12 genes as “all wild-type”; theoretically, these patients should respond to anti-EGFR therapy^23,24^.

### Statistical analysis

Statistical analyses were performed with IBM SPSS Statistics 22 (IBM Japan, Inc., Tokyo, Japan). Five-year OS rates were estimated using the Kaplan-Meier method. The log-rank test was used to assess for significant differences between subgroups by univariate analysis. To investigate independent prognostic factors for OS, factors with a *P*-value of less than 0.05 in univariate analyses were entered into a multivariate analysis. The Cox proportional hazards regression model was used to identify factors that were independently associated with OS after surgery. Pearson’s chi-squared test or Fisher’s exact test was used to evaluate the associations between clinicopathological characteristics and genetic alterations evaluated using CGS. *P*-values less than 0.05 were considered statistically significant.

## Results

### Genetic alterations detected with CGS

CGS of the 415-gene panel successfully detected genetic alterations in all 111 patients, and identified 31 genes altered at a frequency of more than 5% (Fig. 1B). Of the 31 genes, *APC* was the most frequently altered gene, in 92 of 111 patients (83%), followed by *TP53* (77%), *PTEN* (36%), and *KRAS* (35%). Two of 111 patients (2%) were MSI-H.

### Association between Metastatic sites and genetic alterations

Of the 111 patients, metastasis to the liver, lung, peritoneum, and distant lymph nodes at initial assessment were identified in 88, 34, 22, and 21 patients, respectively (Supplementary Table 1). Metastasectomy was performed for 49 of 111 patients. Among the 49 patients, simultaneous metastasectomy was performed for 23 patients, and metachronous metastasectomy was performed for 26 patients. Typically, chemotherapy was administered according to the guidelines^3,7,25,26^. Median number of lines before resection of metastasis was 1 line (range: 1–4 lines). In 31 genes altered at a frequency of more than 5%, liver metastasis was significantly associated with *BRAF* wild-type (*P* = 0.026); lung metastasis was significantly associated with *APC* wild-type (*P* = 0.030); peritoneal metastasis was not significantly associated with any genetic alterations; distant lymph node metastasis was significantly associated with *ATM* mutant-type and *TP53* mutant-type (*P* = 0.010 and *P* = 0.024, respectively).

### Factors influencing OS after primary tumor resection

**Figure 2:**
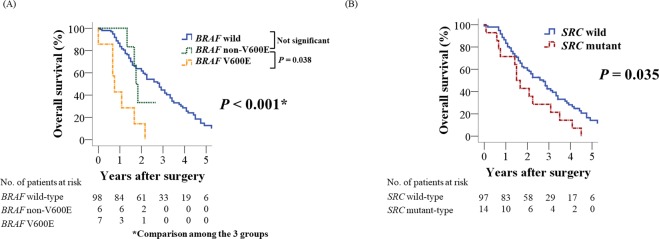
Overall survival after primary tumor resection according to *BRAF* mutation status (**A**). Overall survival according to *SRC* mutation status (**B**).

Of 111 patients, 81 patients were died of cancer while 4 patients were died of other causes. The OS rate after primary tumor resection in 111 patients was 84.6% at one year, 40.8% at three years, and 11.6% at five years. Forty-five of the 111 patients had R0 resection at both primary and metastatic sites (Fig. 1A). Of these, 11 patients had undergone conversion surgery after systemic chemotherapy (Fig. 1A). Univariate analyses revealed that histopathological grade 3, N1, 2 category, *BRAF* V600E mutation (Fig. 2A, Supplementary Fig. 1A), and *SRC* mutation (Fig. 2B, Supplementary Fig. 1B) were associated with worse OS (Table 1) and R0 resection was associated with better OS (Table 1). Regarding *BRAF* non-V600E mutations, OS rate of six patients with *BRAF* non-V600E mutations showed no significant difference compared to *BRAF* wild-type (Fig. 2A). Multivariate analysis identified histopathological grade 3, R0 resection, *BRAF* V600E mutation, and *SRC* mutation as independent prognostic factors for OS (*P* = 0.041, *P* = 0.013, *P* = 0.005, and *P* = 0.023, respectively; Table 1).

### Stratification by BRAF V600E and SRC mutation status in Stage IV CRC

**Figure 3:**
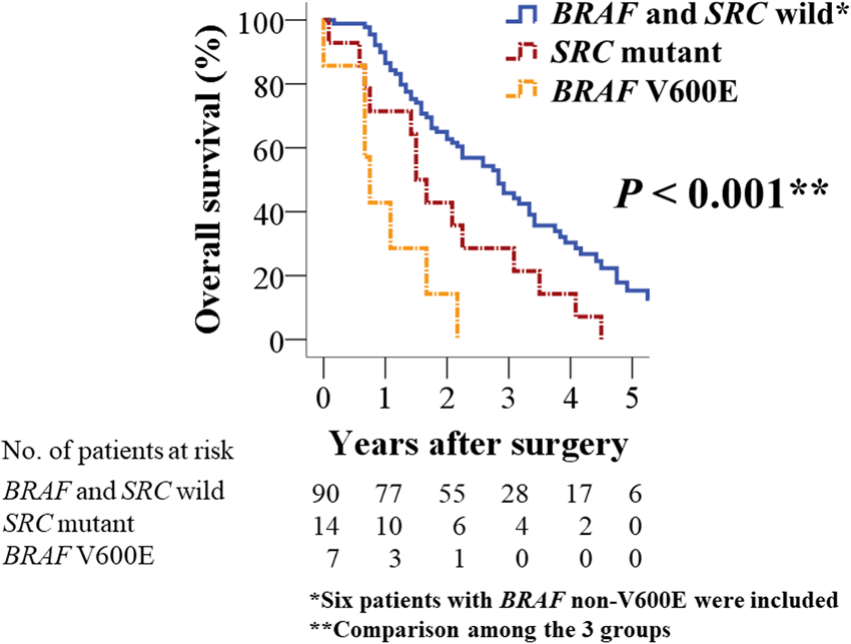
Overall survival after primary tumor resection according to *BRAF* V600E and *SRC* mutation status.

*BRAF* mutations were identified in 13 patients (seven with V600E and six with a non-V600E mutation), and an *SRC* mutation was identified in 14 patients (13 with amplification and one with deletion). *BRAF* V600E and *SRC* mutations were mutually exclusive. Stratification of the 111 patients according to *BRAF* V600E and *SRC* mutation status found patients wild-type for both having significantly better OS (*P* < 0.001; Fig. 3) (Supplementary Figs 2 and 3). In terms of clinicopathological characteristics, *SRC* mutation was significantly associated with left-sided tumor and liver metastasis compared to *BRAF* V600E mutation (*P* = 0.016 and *P* = 0.025, respectively; Table 2).

**Table 2:** Clinicopathological characteristics according to *BRAF* V600E and *SRC* mutation status.

Variable	Modality	*BRAF* V600E and *SRC* status				
		Both *BRAF* V600E and *SRC* wild-type^a^ (N = 90)	*BRAF* V600E (N = 7)	*SRC* mutant (N = 14)	*P*-value^b^	*P*-value^c^
Age (years)	<65	52 (47)	3 (3)	7 (6)	0.667	0.999
	≥65	38 (34)	4 (4)	7 (6)		
Sex	Male	54 (49)	2 (2)	10 (9)	0.164	0.159
	Female	36 (32)	5 (4)	4 (4)		
Tumor size (mm)	<50	33 (30)	0 (0)	3 (3)	0.088	0.521
	≥50	57 (51)	7 (6)	11 (10)		
Pre-operative CEA (ng/ml)	<200	69 (62)	7 (6)	9 (8)	0.190	0.123
	≥200	21 (19)	0 (0)	5 (4)		
Primary tumor site	Right-sided	25 (23)	6 (5)	3 (3)	**0.004**	**0.016**
	Left-sided	65 (58)	1 (1)	11 (10)		
Histopathological grading	G1, 2	67 (60)	1 (1)	10 (9)	**0.004**	**0.024**
	G3	23 (21)	6 (5)	4 (4)		
T category	T2, 3	44 (40)	1 (1)	2 (2)	**0.015**	0.999
	T4	46 (41)	6 (5)	12 (11)		
N category	N0	13 (12)	0 (0)	2 (2)	0.558	0.533
	N1, 2	77 (69)	7 (6)	12 (11)		
M category	M1a	47 (42)	3 (3)	6 (5)	0.742	0.999
	M1b	43 (39)	4 (4)	8 (7)		
Liver metastasis	Absent	18 (16)	4 (4)	1 (1)	**0.027**	**0.025**
	Present	72 (65)	3 (3)	13 (12)		
Lung metastasis	Absent	63 (57)	4 (4)	10 (9)	0.764	0.638
	Present	27 (24)	3 (3)	4 (4)		
Peritoneal metastasis	Absent	74 (67)	4 (4)	11 (10)	0.273	0.354
	Present	16 (14)	3 (3)	3 (3)		
Liver-limited metastasis	Absent	51 (46)	6 (5)	7 (6)	0.269	0.174
	Present	39 (35)	1 (1)	7 (6)		
Number pf metastatic sites	1	51 (46)	5 (4)	8 (7)	0.748	0.656
	≥2	39 (35)	2 (2)	6 (5)		
Residual tumor	R0	41 (37)	1 (1)	3 (3)	0.080	0.593
	R2	49 (44)	6 (5)	11 (10)		

^a^Six patients with *BRAF* non-V600E mutation were included.

^b^The three groups (*BRAF* and *SRC* wild-type, *BRAF* V600E, *SRC* mutant) were compared using a Pearson’s chi-squared test.

^c^*BRAF* V600E versus *SRC* mutant were compared using a Fisher’s exact test (two-tailed).

### Comparison of genetic alterations between initially resectable and initially unresectable groups

Thirty-seven and 74 patients were diagnosed as initially resectable and initially unresectable, respectively (Fig. 1A). No significant differences were observed between the two groups in genetic alterations altered at frequency of more than 10%.

### Genetic alterations of initially resectable group

Of 37 patients who were diagnosed as initially resectable metastatic disease, three patients did not undergo R0 resection because of progressive disease after preoperative chemotherapy; *ARID1A* and *ATM* mutations were significantly associated with progressive disease (*P* = 0.026 and *P* = 0.042, respectively), however, no significant differences were observed in the other genetic alterations at frequency of more than 10%. Regarding MSI status, one of the three patients who showed progressive disease after preoperative chemotherapy had MSI-H tumor.

### Comparison of genetic alterations between conversion and non-conversion groups in initially unresectable patients

**Figure 4:**
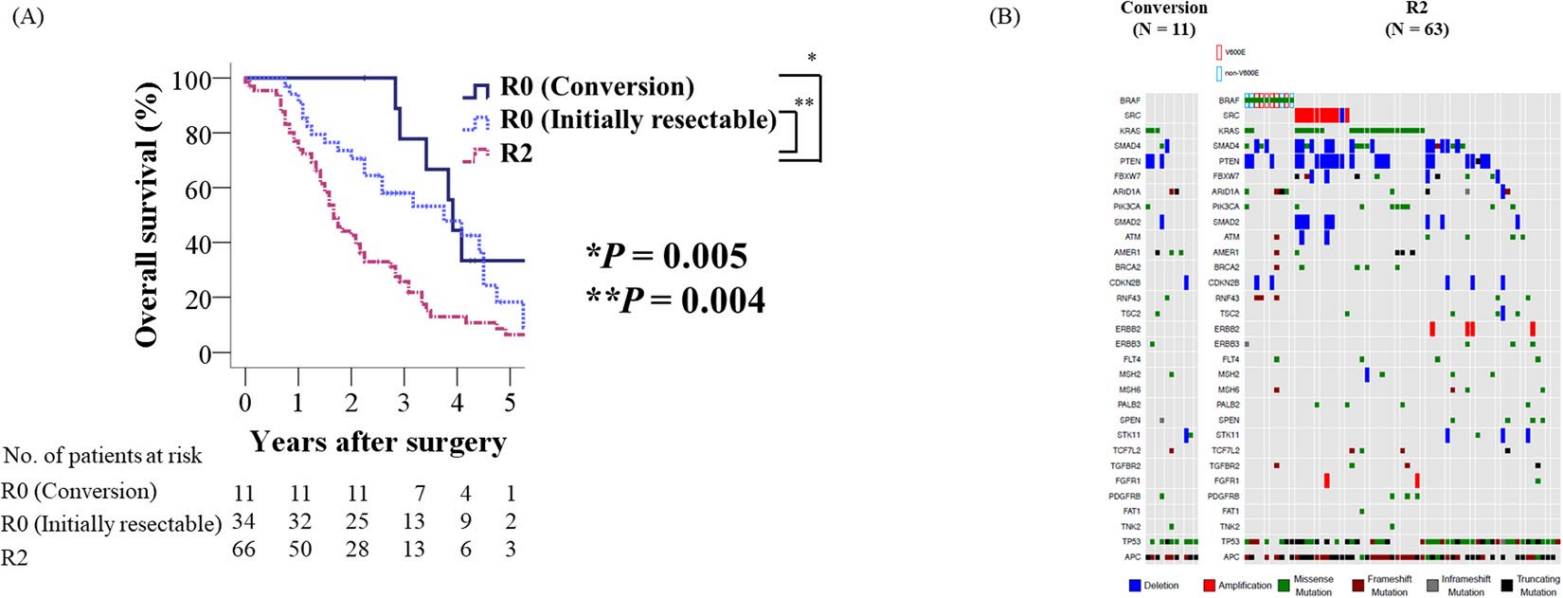
Overall survival according to residual tumor status and conversion surgery (**A**). Oncoprint of conversion surgery group (N = 11) and that of the R2 group (N = 63) in patients with initially unresectable distant metastasis (**B**).

Of 74 patients who were diagnosed as initially unresectable metastatic disease, 11 patients underwent conversion surgery (Fig. 1A, Table 3) and had significantly better OS than R2 patients (*P* = 0.004; Fig. 4A). Regarding the association between metastatic sites and conversion surgery, 8 of 29 patients (28%) with liver-limited disease received conversion surgery, while only 3 of 45 patients (7%) with the other initially unresectable metastases received conversion surgery. *BRAF* or *SRC* mutations were completely absent in the 11 R0 patients that underwent conversion surgery compared with 20 of the 63 R2 patients harboring a mutation in one of these genes (*P* = 0.023; Fig. 4B), however, no significant differences were observed in the other genetic alterations at frequency of more than 10%.

**Table 3:** Clinical course of patients who underwent conversion surgery.

	Age	Sex	Primary site	Initial metastatic sites	Systemic therapy before conversion surgery	Objective response according to RECIST 1.1	Reason that makes the disease resectable	*BRAF* V600E and *SRC* status	Genetic alterations of EGFR pathway^a^	Pattern of failure after conversion surgery	Months after primary tumor resection	Alive or death
1	53	F	Rectosigmoid	Liver	FOLFOX + Pmab	−72%	Significant shrinkage of liver metastases	All wild-type	All wild-type	Lung	27	Alive (NED)^b^
2	59	F	Sigmoid	Liver	FOLFOX + Pmab	−71%	Significant shrinkage of liver metastases	All wild-type	All wild-type	Liver	27	Alive (Tumor bearing)
3	66	F	Rectosigmoid	Liver	XELOX + Bmab	−62%	Significant shrinkage of liver metastases	All wild-type	All wild-type	Liver	52	Alive (NED)^c^
4	50	F	Sigmoid	Liver	XELOX + Bmab	−44%	Significant shrinkage of liver metastases	All wild-type	Mutant	Liver	51	Alive (Tumor bearing)
5	48	F	Sigmoid	Liver and distant LN	FOLFOX + Bmab	−38%	Significant shrinkage of liver metastases (especially near the inferior vena cava)	All wild-type	All wild-type	Liver	49	Dead
6	51	M	Sigmoid	Liver	XELOX + Bmab	−72%	Significant shrinkage of liver metastases	All wild-type	Mutant	Liver	46	Dead
7	56	M	Sigmoid	Liver	XELOX + Bmab	−43%	Significant shrinkage of liver metastases	All wild-type	All wild-type	Liver	35	Dead
8	76	M	Transverse	Liver	FOLFOX + Bmab	−32%	Significant shrinkage of liver metastases (especially near the inferior vena cava)	All wild-type	Mutant	Liver	34	Dead
9	75	F	Sigmoid	Liver	XELOX + Bmab	−45%	Significant shrinkage of liver metastases	All wild-type	All wild-type	Lung	41	Dead
10	78	F	Sigmoid	Liver and lung	FOLFOX	−28%	Significant shrinkage of liver metastases	All wild-type	All wild-type	Liver and peritoneum	72	Dead
11	45	M	Ascending	Liver and peritoneum	FOLFOX	−40%	Significant shrinkage of liver metastases	All wild-type	Mutant	Liver and lung	47	Dead

*Bmab* Bevacizumab, *FOLFOX* 5FU + Leucovorin + Oxaliplatin, *Pmab* Panitumumab, *LN* lymph node, *NED* no evidence of disease, *XELOX* XELODA + Oxaliplatin.

^a^Genetic alterations of EGFR pathway: Genetic alterations of TK receptors (*ERBB2*, *MET*, *EGFR*, *FGFR1*, and *PDGFRA*), MEK/ERK pathway (*KRAS*, *NRAS*, *HRAS*, *BRAF*, and *MAPK2K1*), and PI3K pathway (*PTEN* and *PIK3CA*).

^b^This patient underwent metastasectomies three times (lung, liver, liver) after conversion surgery.

^c^This patient underwent metastasectomies three times (liver, liver, liver) after conversion surgery.

### Genetic alterations in EGFR pathway and response to anti-EGFR therapy

**Figure 5:**
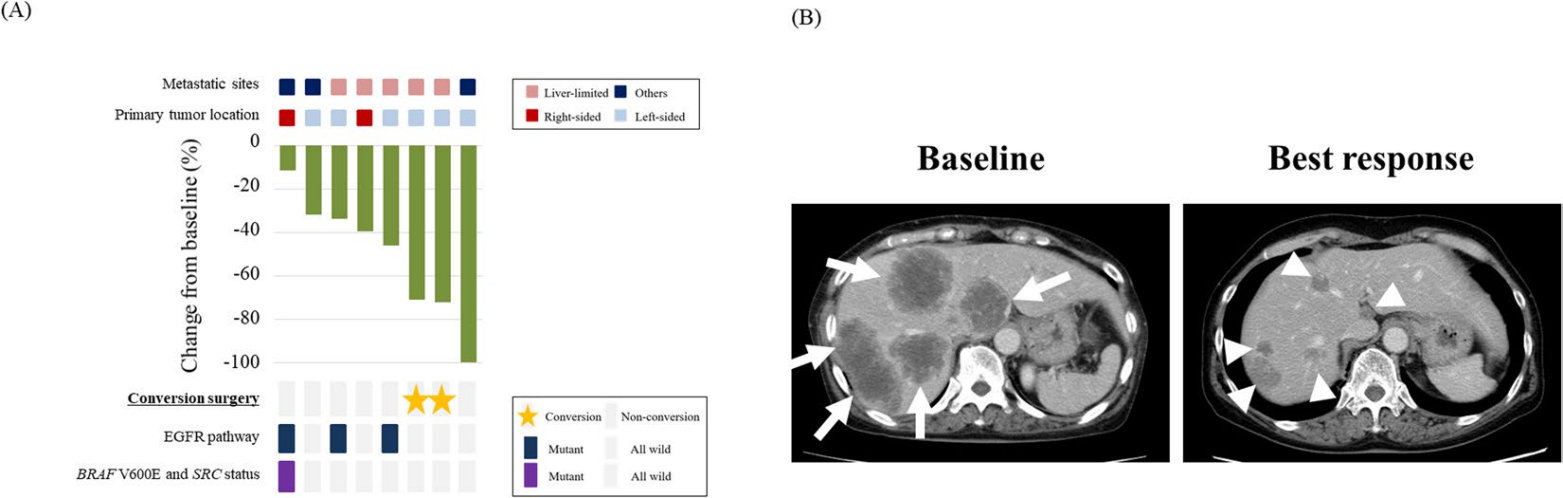
Response to anti-EGFR therapy for patients with initially unresectable metastatic disease. Waterfall plot for 8 patients with initially unresectable disease after anti-EGFR therapy (**A**). Representative case of an “all wild-type” patient who received conversion surgery after anti-EGFR therapy. Arrows show liver metastases at base line. Arrowheads show liver metastases at best response to anti-EGFR therapy (**B**).

In this cohort, 8 of 74 patients with initially unresectable metastatic disease received anti-EGFR therapy with cytotoxic agents (Fig. 1A). CGS revealed that 5 patients were “all wild-type” in EGFR pathway and theoretically should respond to anti-EGFR therapy. Three of the 5 patients (60%) showed significant response to anti-EGFR therapy: 1 patient showed complete response and alive with no evidence of disease, and 2 patients received conversion surgery after anti-EGFR therapy (Fig. 5A,B). Conversely, no patients with “mutant-type” showed complete response to anti-EGFR therapy or received conversion surgery.

## Discussion

The present study had three main findings. First, *BRAF* V600E and *SRC* mutations were identified as independent prognostic factors for OS. Second, *BRAF* V600E and *SRC* mutations were mutually exclusive, and patients with Stage IV CRC were stratified according to *BRAF* V600E and *SRC* mutation status. Third, only patients with wild-type for both *BRAF* V600E and *SRC* mutations underwent R0 conversion surgery. These results imply that *BRAF* V600E and *SRC* mutations are important molecular markers in Stage IV CRC.

CGS can detect numerous important genetic alterations in many solid cancers^8^. Genetic alterations in the MEK/ERK pathway, such as in *KRAS*, *NRAS*, and *BRAF* V600E, are benchmarks used to determine treatment strategies for patients with metastatic CRC. National Comprehensive Cancer Network (NCCN) guidelines state that all patients with metastatic CRC should have tumor tissue genotyped for *KRAS*, *NRAS*, and *BRAF* V600E mutations, and any patient with a known *KRAS* or *NRAS* mutation should not be treated with anti-EGFR therapy such as cetuximab and panitumumab^7^. AJCC 8^th^ edition states *KRAS*, *NRAS*, and *BRAF* V600E mutations are poor prognostic factors in CRC (level II evidence)^2^. In the present analysis, we identified *BRAF* V600E and *SRC* mutations as independent prognostic factors for OS, which may have the potential to stratify Stage IV CRC and predict conversion surgery after systemic therapy. We suggest CGS be utilized in clinical practice to facilitate precision medicine.

CGS allows the simultaneous detection of *BRAF* V600E mutations as well as *BRAF* non-V600E mutations in a single assay. *BRAF* V600E mutation occurs at approximately 5 to 10%, and is one of the important molecular subtypes of CRC^2,3,7^. On the other hand, *BRAF* non-V600E mutations occur at approximately 1 to 5%, and show different clinicopathological characteristics, such as left-sided tumor and better prognosis, than *BRAF* V600E mutation^27–30^. Hence, we separately evaluated *BRAF* V600E and non-V600E mutations in this analysis.

To the best of our knowledge, this is the first report of clinical significance of *SRC* mutation in CRC using CGS. Previous reports demonstrated that activating mutation of *SRC* was found in 12% of CRC patients^31,32^, and indicated that *SRC* mutation is associated with distant metastasis and drug resistance in CRC^14–18^. In this analysis, we reported that 14 of 111 patients (13%) had *SRC* mutation, which was significantly associated with left-sided tumor and liver metastasis compared to *BRAF* V600E mutation. Multivariate analysis revealed that *SRC* mutations were independent prognostic factors on OS in Stage IV CRC. Moreover, *BRAF* V600E and *SRC* mutations were mutually exclusive. Taken together, we consider that *SRC* mutation might be an important molecular subtype in CRC.

Although this analysis found patients with *BRAF* V600E or *SRC* mutations had a poorer prognosis and seem poor candidates for conversion surgery, *BRAF* V600E and *SRC* mutations are regarded as druggable. In melanoma, the *BRAF* V600E mutation is the target of the *BRAF*-mutant inhibitor vemurafenib^33^. However, *BRAF* V600E mutant CRCs are not sensitive to *BRAF*-mutant inhibitors due to the feedback reaction of EGFR^34^. As such, clinical trials are planned for metastatic CRC using combinations of *BRAF*-mutant inhibitors, MEK and EGFR inhibition^35,36^. SRC activity is considered to play a key role in CRC development and metastasis, and there are several trials using SRC inhibitors such as dasatinib^37,38^. Thus, targeting BRAF V600E and SRC may represent a future treatment strategy for Stage IV CRC, which has the possibility to improve OS and rate of conversion surgery in these dismal molecular subtypes.

Emerging CGS technologies enable the detection of numerous genetic mutations in a single assay, and we can now analyze the clinical significance regarding not only “single mutation” such as *BRAF* V600E and *SRC*, but also various combinations of genetic alterations using CGS. Recently, clinical impact of “double mutation” has been reported in patients with CRC. Yamashita *et al*. reported that double mutation of *APC* and *PIK3CA* was associated with response to preoperative chemotherapy and poor survival for colorectal liver metastasis^39^. Chun *et al*. reported that double mutation of *RAS* and *TP53* was associated with shorter OS after hepatectomy for colorectal liver metasstasis^40^. Chow *et al*. reported that double mutation of *KRAS* and *TP53* was associated with lymph node metastasis in Stage II/III rectal cancer patients who received chemoradiotherapy followed by total mesorectal excision^41^. Although we did not find clinical significance of these double mutations (*APC* and *PIK3CA*, *RAS* and *TP53*, *KRAS* and *TP53*) in our cohort, we consider that, not only “single mutation”, but also “double mutation” might be an important concept in the era of precision medicine.

To achieve conversion surgery for initially unresectable patients, a regimen leading to high response rates and/or a large tumor size reduction is recommended, and a cytotoxic doublet plus an anti-EGFR antibody is applied for patients with *RAS* wild-type disease^3^. Although *RAS* mutations are established biomarkers of efficacy to anti-EGFR therapy, anti-EGFR therapy is not effective for all patients with a *RAS* wild-type tumor. Genetic alterations in TK receptors, the RAS pathway (other than *KRAS* and *NRAS* mutations), and the PI3K pathway are other possible mechanisms of resistance to anti-EGFR therapy^25,26^. In the present analysis, we defined patients who had no alterations in any of the 12 genes as “all wild-type”, and demonstrated that “all wild-type” in the EGFR pathway might be a predictor for significant response to anti-EGFR therapy and subsequent conversion surgery. Thus, we consider that, in addition to *BRAF* and *SRC* mutations, genetic alterations in EGFR pathway are also important for precision medicine of Stage IV CRC.

This analysis has several limitations. First, it was a retrospective analysis performed at two institutions and included a relatively small number of patients. Second, this analysis included patients who underwent various systemic therapies with or without targeted therapy. Third, this analysis did not include the patients who did not undergo primary tumor resection. Fourth, this analysis did not include patients whose treatment was tailored based upon their tumor’s individual genetic alterations. As we are now in the era of precision medicine, future analyses need to assess the value of CGS in patients whose treatment has been tailored to their tumor’s genetic alterations.

In conclusion, *BRAF* V600E and *SRC* mutations are important molecular markers which can predict prognosis and conversion surgery in Stage IV CRC.

## Supplementary information

Dataset 1

Dataset 2

## References

[1] World Health Organization. GLOBOCAN 2012: Estimated Cancer Incidence, Mortality and Prevalence Worldwide in 2012, http://globocan.iarc.fr/Pages/fact_sheets_population.aspx (2012).

[2] Amin, M. B. *et al*. AJCC Cancer Staging Manual. 8th edition (Springer, 2017).

[3] Van Cutsem E (2016). ESMO consensus guidelines for the management of patients with metastatic colorectal cancer. Ann Oncol..

[4] Kattan MW (2016). American Joint Committee on Cancer acceptance criteria for inclusion of risk models for individualized prognosis in the practice of precision medicine. CA Cancer J Clin..

[5] Asare EA, Washington MK, Gress DM, Gershenwald GE, Greene FL (2015). Improving the quality of cancer staging. CA Cancer J Clin..

[6] Allegra CJ (2009). American Society of Clinical Oncology provisional clinical opinion: testing for KRAS gene mutations in patients with metastatic colorectal carcinoma to predict response to anti-epidermal growth factor receptor monoclonal antibody therapy. J Clin Oncol..

[7] National Comprehensive Cancer Network. NCCN clinical practice guidelines in oncology-rectal cancer (version 1. 2018), https://www.nccn.org/professionals/physician_gls/pdf/colon.pdf (2018). 10.6004/jnccn.2018.0006 29439178

[8] Cancer Genome Atlas Network (2012). Comprehensive molecular characterization of human colon and rectal cancer. Nature..

[9] Gavin PG (2012). Mutation profiling and microsatellite instability in stage II and III colon cancer: an assessment of their prognostic and oxaliplatin predictive value. Clin Cancer Res..

[10] Weiser MR, Jarnagin WR, Saltz LB (2013). Colorectal cancer patients with oligometastatic liver disease: what is the optimal approach?. Oncology (Williston Park)..

[11] Adam R (2004). Rescue surgery for unresectable colorectal liver metastases downstaged by chemotherapy: a model to predict long-term survival. Ann Surg..

[12] Okuno M (2017). Does response rate of chemotherapy with molecular target agents correlate with the conversion rate and survival in patients with unresectable colorectal liver metastases?: A systematic review. Eur J Surg Oncol..

[13] Yeatman TJ (2004). A renaissance for SRC. Nat Rev Cancer..

[14] Chen J, Elfiky A, Han M, Chen C, Saif MW (2014). The role of Src in colon cancer and its therapeutic implications. Clin Colorectal Cancer..

[15] Termuhlen PM, Curley SA, Talamonti MS, Saboorian MH, Gallick GE (1993). Site-specific differences in pp60c-src activity in human colorectal metastases. J Surg Res..

[16] Aligayer H (2002). Activation of Src kinase in primary colorectal carcinoma: an indicator of poor clinical prognosis. Cancer..

[17] Griffiths GJ (2004). Expression of kinase-defective mutants of c-Src in human metastatic colon cancer cells decreases Bcl-xL and increases oxaliplatin- and Fas-induced apoptosis. J Biol Chem..

[18] Kopetz S (2009). Synergistic activity of the SRC family kinase inhibitor dasatinib and oxaliplatin in colon carcinoma cells is mediated by oxidative stress. Cancer Res..

[19] Nagahashi M (2016). Genomic landscape of colorectal cancer in Japan: clinical implications of comprehensive genomic sequencing for precision medicine. Genome Med..

[20] Shimada Y (2017). Utility of comprehensive genomic sequencing for detecting HER2-positive colorectal cancer. Hum Pathol..

[21] Shimada Y (2017). Comprehensive genomic sequencing detects important genetic differences between right-sided and left-sided colorectal cancer. Oncotarget..

[22] Edge, S. B. *et al*. AJCC Cancer Staging Manual. 7th edition (Springer, 2010).

[23] Bertotti A (2015). The genomic landscape of response to EGFR blockade in colorectal cancer. Nature..

[24] Bertotti A (2011). A molecularly annotated platform of patient-derived xenografts (“xenopatients”) identifies HER2 as an effective therapeutic target in cetuximab-resistant colorectal cancer. Cancer Discov..

[25] Watanabe T (2018). Japanese Society for Cancer of the Colon and Rectum (JSCCR) guidelines 2016 for the treatment of colorectal cancer. Int J Clin Oncol..

[26] Japanese Society for Cancer of the Colon and Rectum. Japanese classification of colorectal carcinoma. 2nd English ed. (Kanehara & Co, 2009).

[27] Cremolini C (2015). BRAF codons 594 and 596 mutations identify a new molecular subtype of metastatic colorectal cancer at favorable prognosis. Ann Oncol..

[28] Jones JC (2017). (Non-V600) BRAF Mutations Define a Clinically Distinct Molecular Subtype of Metastatic Colorectal Cancer. J Clin Oncol..

[29] Shinozaki E (2017). Clinical significance of BRAF non-V600E mutations on the therapeutic effects of anti-EGFR monoclonal antibody treatment in patients with pretreated metastatic colorectal cancer: the Biomarker Research for anti-EGFR monoclonal Antibodies by Comprehensive Cancer genomics (BREAC) study. Br J Cancer..

[30] Shimada Y (2018). Clinical significance of BRAF non-V600E mutations in colorectal cancer: A retrospective study of two institutions. J Surg Res..

[31] Irby RB (1999). Activating SRC mutation in a subset of advanced human colon cancers. Nat Genet..

[32] Irby RB, Yeatman TJ (2000). Role of Src expression and activation in human cancer. Oncogene..

[33] Sosman JA (2012). Survival in BRAF V600-mutant advanced melanoma treated with vemurafenib. N Engl J Med..

[34] Prahallad A (2012). Unresponsiveness of colon cancer to BRAF(V600E) inhibition through feedback activation of EGFR. Nature..

[35] Corcoran RB (2015). Combined BRAF and MEK Inhibition With Dabrafenib and Trametinib in BRAF V600-Mutant Colorectal Cancer. J Clin Oncol..

[36] Hyman DM (2015). Vemurafenib in Multiple Nonmelanoma Cancers with BRAF V600 Mutations. N Engl J Med..

[37] Kopetz S (2014). Src activity is modulated by oxaliplatin and correlates with outcomes after hepatectomy for metastatic colorectal cancer. BMC Cancer..

[38] Parseghian CM (2017). Dual Inhibition of EGFR and c-Src by Cetuximab and Dasatinib Combined with FOLFOX Chemotherapy in Patients with Metastatic Colorectal Cancer. Clin Cancer Res..

[39] Yamashita, S. *et al*. APC and PIK3CA Mutational Cooperativity Predicts Pathologic Response and Survival in Patients Undergoing Resection for Colorectal Liver Metastases. *Ann Surg*., 10.1097/SLA.0000000000002245 (2017). 10.1097/SLA.0000000000002245 28379870

[40] Chun, Y. S. *et al*. Deleterious Effect of RAS and Evolutionary High-risk TP53 Double Mutation in Colorectal Liver Metastases. *Ann Surg*., 10.1097/SLA.0000000000002450 (2017). 10.1097/SLA.0000000000002450 PMC7462436 28767562

[41] Chow OS (2016). KRAS and Combined KRAS/TP53 Mutations in Locally Advanced Rectal Cancer are Independently Associated with Decreased Response to Neoadjuvant Therapy. Ann Surg Oncol..

