# Supplementary material for: BRAF V600E and SRC mutations as molecular markers for predicting prognosis and conversion surgery in Stage IV colorectal cancer
Source: Sci Rep. 2019 Feb 21;9:2466. doi: 10.1038/s41598-019-39328-6 (PMC6384937; doi:10.1038/s41598-019-39328-6)
Supplement: Supplementary file 1 — Dataset 1 [file 41598_2019_39328_MOESM1_ESM.docx]

Title:

***BRAF* V600E and *SRC* mutations as molecular markers for predicting prognosis and conversion surgery in Stage IV colorectal cancer**

Yoshifumi Shimada MD, PhD^a^, Yusuke Muneoka MD, PhD^a^, Masayuki Nagahashi MD, PhD^a^, Hiroshi Ichikawa MD, PhD^a^, Yosuke Tajima MD, PhD^a^, Yuki Hirose MD, PhD^a^, Takuya Ando MD^a^, Masato Nakano MD, PhD^a^, Jun Sakata MD, PhD^a^, Hitoshi Kameyama MD, PhD^a^, Yasumasa Takii MD, PhD^b^, Yiwei Ling PhD^c^, Shujiro Okuda PhD^c^, Kazuaki Takabe MD, PhD, FACS ^a,d-g^, Toshifumi Wakai MD, PhD, FACS^a^

^a^ Division of Digestive and General Surgery, Niigata University Graduate School of Medical and Dental Sciences, Niigata, Japan

^b^ Department of Surgery, Niigata Cancer Center Hospital, Niigata, Japan

^c^ Division of Bioinformatics, Niigata University Graduate School of Medical and Dental Sciences, Niigata, Japan

^d^ Division of Breast Surgery, Roswell Park Comprehensive Cancer Center, Elm & Carlton Streets, Buffalo, NY 14263, USA.

^e^ Department of Surgery, University at Buffalo Jacobs School of Medicine and Biomedical Sciences, The State University of New York, Buffalo, NY, USA

^f^ Department of Breast Surgery and Oncology, Tokyo Medical University, Tokyo, Japan

^g^ Department of Surgery, Yokohama City University, Yokohama, Japan

**Correspondence:**

Yoshifumi Shimada, Division of Digestive and General Surgery, Niigata University Graduate School of Medical and Dental Sciences, 1-757 Asahimachi-dori, Chuo-ku, Niigata City, Niigata 951-8510, Japan, Phone: +81-25-227-2228, Fax: +81-25-227-0779, E-mail address: [shimaday@med.niigata-u.ac.jp](mailto:shimaday@med.niigata-u.ac.jp)

Toshifumi Wakai, Division of Digestive and General Surgery, Niigata University Graduate School of Medical and Dental Sciences, 1-757 Asahimachi-dori, Chuo-ku, Niigata City, Niigata 951-8510, Japan, Phone: +81-25-227-2228, Fax: +81-25-227-0779, E-mail address: [wakait@med.niigata-u.ac.jp](mailto:shimaday@med.niigata-u.ac.jp)


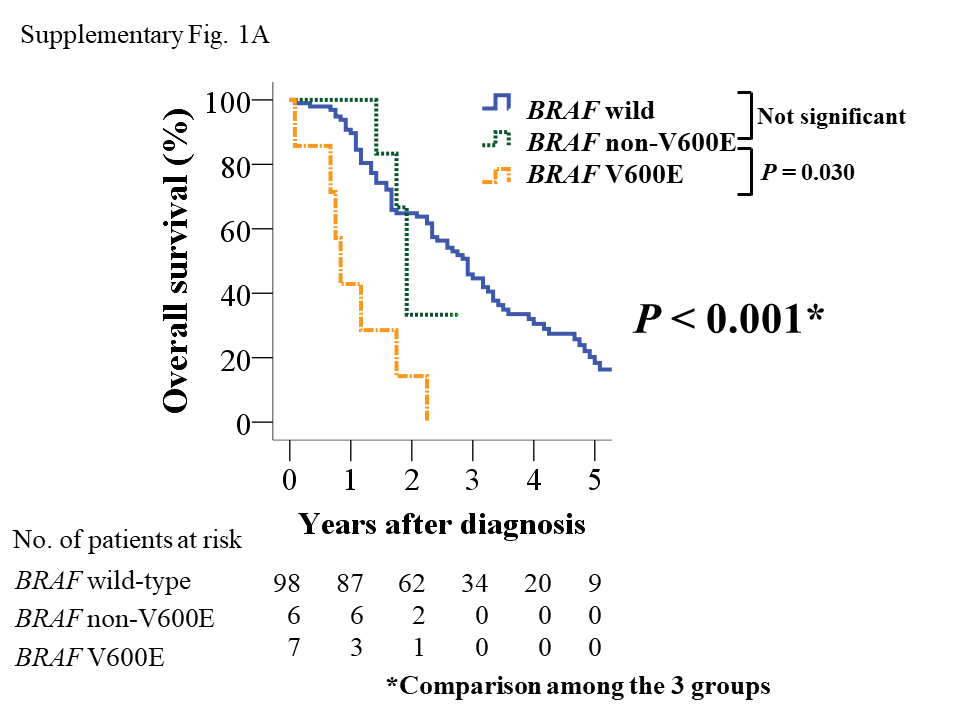


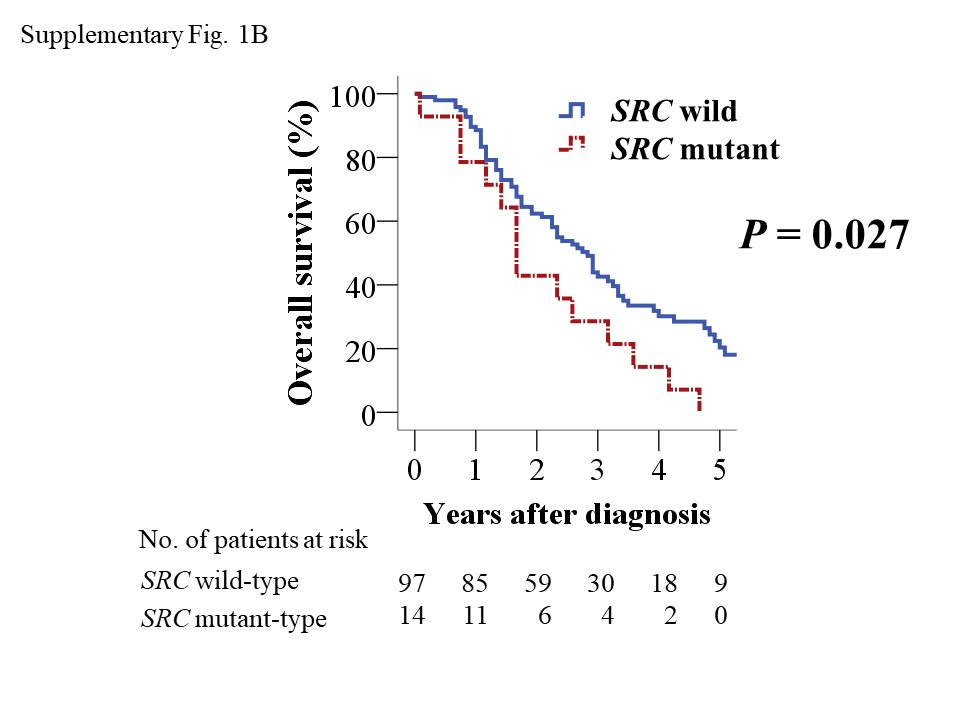


**Supplementary Figure 1.**

Overall survival after diagnosis according to *BRAF* mutation status (**A**). Overall survival after diagnosis according to *SRC* mutation status (**B**).


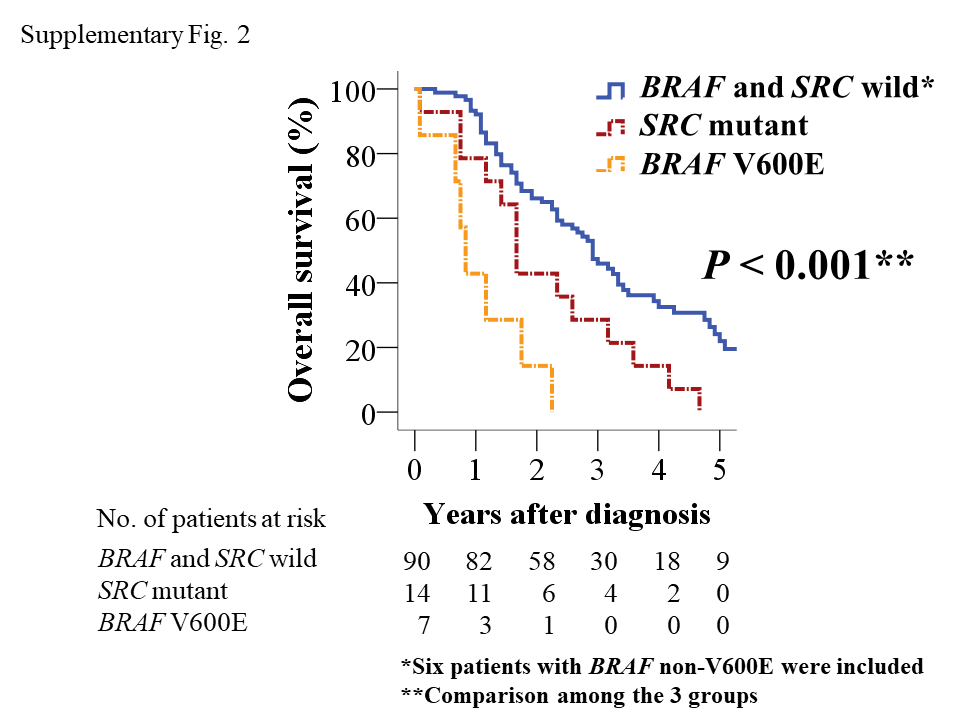


**Supplementary Figure 2.**

Overall survival after diagnosis according to *BRAF* V600E and *SRC* mutation status.


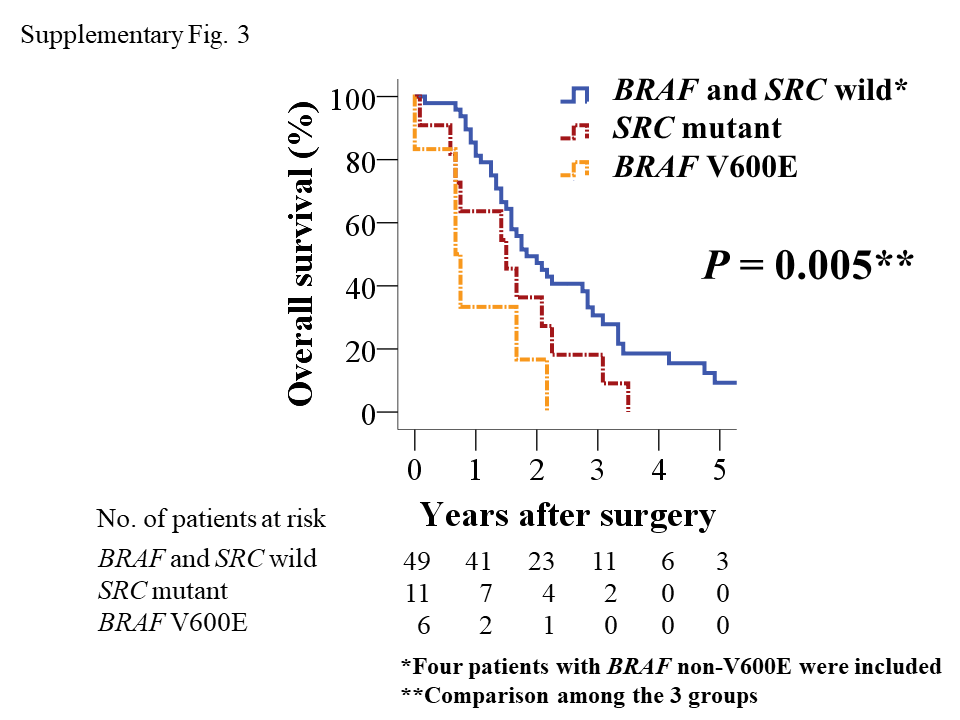


**Supplementary Figure 3.**

Overall survival after surgery according to *BRAF* V600E and *SRC* mutation status in R2 group (N = 66).
